# Hygroscopic additive-modified magnesium sulfate thermochemical material construction and heat transfer numerical simulation for low temperature energy storage

**DOI:** 10.1039/d2ra00344a

**Published:** 2022-03-21

**Authors:** Shi-Jie Li, Xiang-Yu Yang, Li-Sheng Deng, Yong-Chun Fu, Ming-Jun Pang, Ti Dong, Yi-Song Yu, Ling-Na Su, Shang Jiang

**Affiliations:** Institute of Carbon Materials Science, Shanxi Datong University Datong 037009 P. R. China jiangshang3714@163.com; Key Laboratory of Renewable Energy, Guangdong Provincial Key Laboratory of New and Renewable Energy Research and Development, Guangzhou Institute of Energy Conversion, Chinese Academy of Sciences No. 2 Nengyuan Rd., Wushan, Tianhe District Guangzhou 510640 P. R. China; School of Materials Science and Engineering, Taiyuan University of Technology Taiyuan 030024 P. R. China; Guangdong Intelligent Filling Technology Limited Company No. 63 (F3) 5, Zone C, Sanshui Industrial Park Foshan Guangdong 528137 P. R. China

## Abstract

In this research, the core objective is to explore the effect of super-absorbent polymer material (poly(sodium acrylate)) on the heat storage performance of magnesium sulfate and to investigate the heat transfer behavior of 13X-zeolite, nano-aluminum oxide (nano-Al_2_O_3_) and poly(sodium acrylate) modified magnesium sulfate in a reactor. Finally it provides support for future material and reactor design. All characterizations and performance tests were done in the laboratory and a numerical simulation method was used to investigate the heat transfer behavior of the reactor. Through hydrothermal treatment, bulk MgSO_4_·6H_2_O was changed into nanoparticles (200–500 nm) when composited with poly(sodium acrylate), 13X-zeolite and nano-Al_2_O_3_. Among these materials, MgSO_4_·6H_2_O shows the highest activation energy (36.8 kJ mol^−1^) and the lowest energy density (325 kJ kg^−1^). The activation energy and heat storage energy density of nano-Al_2_O_3_ modified composite material MA-1 are 28.5 kJ mol^−1^ and 1305 kJ kg^−1^, respectively. Poly(sodium acrylate) modified composite material, MPSA-3, shows good heat storage energy density (1100 kJ kg^−1^) and the lowest activation energy (22.3 kJ mol^−1^) due its high water-absorbing rate and dispersing effect. 13X-zeolite modified composite material MZ-2 shows lower activation energy (32.4 kJ mol^−1^) and the highest heat storage density (1411 kJ kg^−1^), which is 4.3 times higher than that of pure magnesium sulfate hexahydrate. According to the heat transfer numerical simulation, hygroscopic additives could prominently change the temperature distribution in the reactor and efficiently release heat to the thermal load side. The experimental and numerical simulation temperatures are similar. This indicates that the result of the numerical simulation is very close to the actual heat transfer behavior. This reactor could output heat at around 50 °C and absorb heat in the range of 100–200 °C. All these results further prove the strategy that thermochemical nanomaterial synthesis technology combined with material-reactor heat transfer numerical simulation is feasible for future material and reactor design.

## Abstract 2

Construction of a high performance hydrophilic magnesium sulfate composite thermal energy storage material and numerical simulation of its heat transfer behavior.

## Graphical abstract

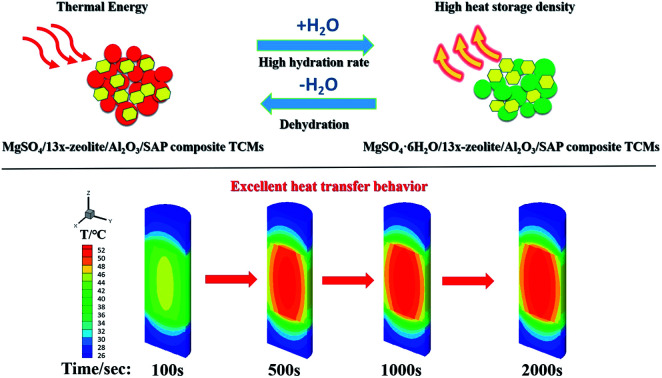

## 1. Introduction

The storage of waste heat or solar energy is an important way to promote the utilization efficiency of renewable and sustainable energy and reduce the consumption of fossil fuels. In achieving this target, various materials with high storage capacity based on the matching system have been designed.^1,2^ These technologies can commonly be divided into three main types: sensible heat storage,^3,4^ latent heat storage^5,6^ and thermochemical heat storage.^7–11^ However, the first two technologies can more easily lose conserved thermal energy, and are therefore not appropriate candidates for long-term heat storage.^12^ Among these technologies, thermochemical heat storage using a reversible chemical reaction with thermal energy change to release and store heat shows the highest efficiency for thermal energy utilization because of its excellent heat storage density.^13^ Large numbers of materials could thus be researched for use in thermochemical heat storage over a wide range of working temperatures.^12–19^ Kubota *et al.*^9,20^ composed a porous carbon and hygroscopic material with lithium hydroxide (LiOH) for low-temperature energy storage and the heat storage performance was obviously improved. This research proves that additive materials could enhance the performance and the heat and mass transfer property of the materials. Pierrès and other researchers^21^ studied the heat and mass transfer mechanism of the hydrated salt strontium chloride monohydrate (SrCl_2_·H_2_O) in an open reactor system. The finite element method was used to construct a two-dimensional model of the reactor. Their results indicate that the water steam partial pressure and reactor inlet pressure have a tremendous influence on the thermal energy storage behavior. Mass transfer is the main parameter for controlling the performance of the open reactor. Malley-Ernewien *et al.*^22^ showed the influence of heat and mass transfer properties, such as pressure loss and temperature distribution, on the construction of a chemical heat storage reactor. This study shows that an increasing number of reaction beds and increasing bed compactness favour an improvement in the performance of a thermal energy storage reactor. Luo *et al.*^23^ listed summaries of the advantages and prospects of salt hydrate thermochemical energy storage, especially metal–organic framework (MOF) materials that are used for salt hydrate-based thermal energy storage. They also showed the importance of heat and mass transfer in the materials and the reactor. For the sake of efficiently recycling low-temperature thermal energy at around 150 °C, inorganic hydrate magnesium sulfate heptahydrate (MgSO_4_·7H_2_O) was selected. It is considered to be an excellent heat storage material for low-temperature thermal energy utilization. It is also non-toxic, low-cost and non-corrosive with potential for green energy applications in buildings. The endothermic/exothermic reaction of magnesium sulfate is also related to magnesium sulfate (MgSO_4_) and water vapour in the atmosphere with high relative humidity and the release of chemical energy. But, the reaction cannot be fully completed.^24^ When MgSO_4_ and water are stored separately, the former thermal energy, which has been converted to chemical bond energy, could be stored long-term.

In order to improve the heat storage performance of MgSO_4_, many researchers have done a lot of work. Posern and Kaps^18^ found that when magnesium chloride (MgCl_2_) was added into MgSO_4_, the water sorption behavior of these binary composite heat storage materials was greatly changed. MgCl_2_ partially replaced MgSO_4_ to reduce the deliquescence relative humidity of the mixture, so as to increase the condensation capacity and energy density. But, meanwhile, the chlorides presented certain corrosivity. Therefore, the mixed ratio of these two salts should be well controlled. Ata Ur Rehman *et al.*^25^ prepared an MgSO_4_/ZnSO_4_ (zinc sulfate) composite material and investigated its heat storage performance. The result showed that the energy density and water adsorption amount of this binary composite were notably enhanced. This may be due to the optimal mixed ratio and better adsorption property compared to each single salt. Hongois *et al.*^26^ used zeolite as an additive for a magnesium sulfate heat storage material. And the heat storage performance was obviously improved. MgSO_4_ could be well dispersed on the zeolite surface due to its porous expanded structure. This type of structure also benefited the thermal energy release and absorbance. Xu *et al.*^27^ investigated the hydration behavior of zeolite–MgSO_4_ composites for heat storage. They found that zeolite–MgSO_4_ materials showed higher heat storage performance and hydration ability than pure zeolite. But the hydration ability greatly decreased when the temperature was higher than 50 °C. It can be seen from the above research that magnesium sulfate hydrate materials still have many shortcomings which need to be solved. Similar to other inorganic hydrates,^28^ both the heat and mass transfer and reaction performance of magnesium sulfate hydrate materials are poor,^24^ which severely restricts the commercial application of magnesium sulfate heptahydrate for heat storage. Consequently, a heat storage material based on MgSO_4_ with excellent water vapor uptake and high thermal conductivity urgently needs to be developed. 13X-zeolite, nano-aluminum oxide and a super-absorbent polymer material, which in this research is poly(sodium acrylate), are chosen as hygroscopic additives for thermal energy utilization. 13X-zeolite and nano-aluminum oxide as excellent porous materials are always used for gas separation, for instance, volatile organic chemicals,^29^ carbon dioxide,^30^ catalysts^31^ and solar cells,^32^ due to their excellent adsorption property and porosity. The super-absorbent polymer (SAP) material, poly(sodium acrylate), has a strong adsorption effect on neighbouring water molecules and is always used for sewage treatment and electrics.^33,34^

In this work, due to the instability of MgSO_4_·7H_2_O at room temperature and atmospheric pressure, it is necessary to heat it to produce stable magnesium sulfate hexahydrate (MgSO_4_·6H_2_O). Meanwhile, the super-absorbent polymer material, poly(sodium acrylate), has rarely been researched for thermochemical heat storage. The heat transfer behavior of poly(sodium acrylate), porous 13X-zeolite and nano-Al_2_O_3_ modified magnesium sulfate in a reactor is not yet well known. Therefore, in order to develop new heat storage materials and further improve reactor design, four types of thermochemical materials were prepared. The heat storage performance was investigated and the heat transfer behavior of the materials in the reactor was numerically simulated.

## 2. Experimental

### 2.1 Materials and methods

**Fig. 1:**
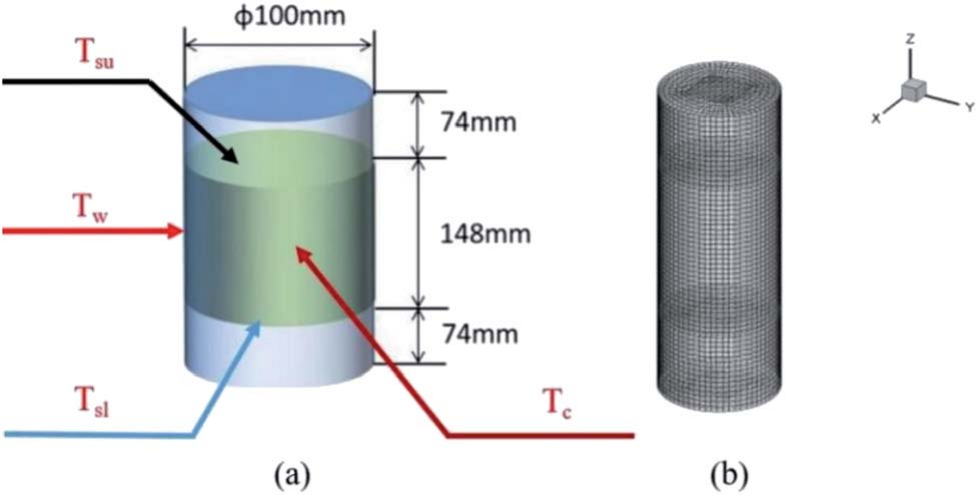
(a) The physical model and (b) heat transfer 3D numerical grid of the reactor with heat storage materials.

The thermochemical materials were prepared by a hydrothermal method using MgSO_4_·7H_2_O (Aladdin, Ltd, purity 99.0%) as the raw material and poly(sodium acrylate) (Shengli Oil Field Changan Group, purity >98.0%), 13X-zeolite (Damao Chemical Reagent Factory, purity 98.0%) and nano-Al_2_O_3_ (Aladdin, Ltd, purity 99.9%) as hygroscopic additives. Firstly, MgSO_4_ solution was prepared by slowly adding 1186 mg of MgSO_4_·7H_2_O into 10 mL of DI water under strong stirring at 25 °C for 5 min and then moved into a 20 mL stainless steel hydrothermal reactor. After that, 785 mg of poly(sodium acrylate) was placed into the reactor also under stirring for 1 h. Finally the reactor was heated to 150 °C and kept there for 8 h. During the hydrothermal process, due to the increase in temperature and pressure in the hydrothermal reactor, the solubility of the materials starts to decrease and reach saturation, and they precipitate from the solution in a crystalline form of the compound type. The materials generate corresponding coordination aggregates through hydrolysis and polycondensation. When the concentration reaches supersaturation, the materials begin to precipitate many crystal nuclei and finally grow into small crystals. By controlling the hydrothermal temperature and time, further growth of crystalline particles is limited and a large number of nanoparticles are eventually formed around the crystal nuclei. Meanwhile, because of the uniform contact between additives and MgSO_4_ solution, nanoparticles could easily be generated and well dispersed on the additive materials. When the obtained material had cooled to room temperature, it was dried at 150 °C for 2 h in a horizontal tubular quartz furnace under Ar buffer gas. After that the material temperature was reduced to 30 °C and it was reacted with water vapor carried with N_2_ flow gas for 30 min. Then all the samples were heated to 40 °C for 24 h, and finally the target products were obtained. All the samples were prepared using the same method. Definitions of the names of the four obtained materials are listed in Table 1. A large number of products were synthesized and collected, which were dried at 150 °C for 2 h. After that the obtained materials (700 g) and water vapor flow were loaded in a stainless-steel cylindrical reactor (*ϕ* 100 mm × *h* 300 mm × *w* 2 mm) at 30 °C. It was equipped with four K-type thermocouples, located at the center, the inner wall, upper surface and lower surface of the materials in the reactor. These thermocouples were used to test the temperature (*T*_c_, *T*_w_, *T*_su_, *T*_sl_) of the above-mentioned four positions, as shown in Fig. 1a.

**Table 1:** Definition of the names of hygroscopic nanoadditive modified magnesium sulfate based thermochemical materials

Sample name	Materials composition
MA-1	MgSO_4_·6H_2_O/Al_2_O_3_
MZ-2	MgSO_4_·6H_2_O/13X-zeolite
MPSA-3	MgSO_4_·6H_2_O/poly(sodium acrylate)
MgSO_4_·6H_2_O	MgSO_4_·6H_2_O

After 30 min the values of the temperatures of *T*_c_, *T*_w_, *T*_su_ and *T*_sl_ of the four materials were recorded. These experimental temperature values were used as a reference for a comparison with the numerical simulation temperatures to verify their correctness (Fig. 8).

### 2.2 Characterization and performance testing

The microstructure was measured by field-emission scanning electron microscopy (SEM, S-4800, Hitachi Limited). X-ray diffraction (XRD) analysis was performed on a D8-Advance X-ray diffractometer (Bruker, Germany) with a Cu target (40 kV, 40 mA). Nitrogen adsorption–desorption was measured at the boiling point of nitrogen (77 K) using a Quantachrome QDS-30 analyzer. The BET surface area and pore structure were measured by nitrogen physisorption under a normal relative pressure of 0.1–1.0. The thermal conductivity of the samples was measured by a TPS2500S thermal conductivity tester (Hot Disk, Sweden). The water vapor adsorption properties were tested using a constant-temperature-and-humidity test box (YNK/TH-150, Suzhou UNIQUE Environmental Test Equipment, China). The energy and mass change of the samples were measured with an STA-449F5 simultaneous thermal analyzer (Netzsch Co., Ltd, Germany). The activation energies of the dehydration reaction of all the samples were calculated by the Kissinger method.^35^ According to this method, the following equation can be obtained based on the reaction rate expression and Arrhenius's equation:1
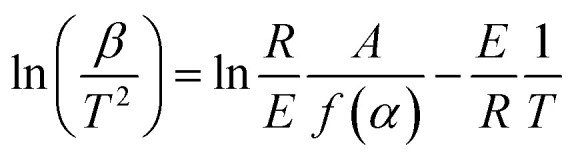
In this equation, *E* is the activation energy [kJ mol^−1^], *β* is the heating rate [K min^−1^], *T* is the peak temperature [K], *R* is the molar gas constant [J (mol^−1^·K^−1^)], *A* is a pre-exponential factor, *α* is the dehydration conversion and *f*(*α*) is a function of dehydration conversion, which here takes a fixed value. During the calculation of activation energy, the heating rates were 5 K min^−1^, 10 K min^−1^, 15 K min^−1^ and 20 K min^−1^, and the activation energy was obtained from the slope (−*E*/*R*) of this equation.

### 2.3 Heat transfer numerical simulation of a magnesium sulfate based composite heat storage material in reactor

For the heat transfer numerical simulation, commercial computational fluid dynamics (CFD) software, fluent, which is based on the finite volume approximation method, was selected and used. Firstly, a stainless-steel cylindrical reactor with a bottom internal diameter of 100 mm, height of 300 mm and wall thickness of 2 mm was constructed as a physical model (Fig. 1a). As shown in Fig. 1a, *T*_c_ and *T*_w_ stood for the core temperature and inner wall temperature, respectively, of the reactor filled with heat storage material. *T*_sl_ and *T*_su_ were the lower surface and upper surface temperature, respectively, of the heat storage material in the reactor. After building the physical model, the heat transfer behavior was simulated according to the hydration reaction in the reactor. A cylindrical reaction region with nitrogen and reactants as a whole system was established (Fig. 1b). The governing equation of the air domain is eqn (2) and the governing equation for the sample region is eqn (3), where *ρ* is the gas density, kg m^−3^; *C*_p_ is the specific heat capacity, J kg^−1^ K^−1^; *T* is the temperature, K; *t* is the time, s; *k* is the thermal conductivity coefficient, W m^−1^ K^−1^;2
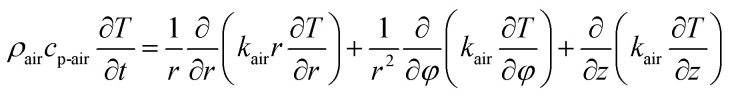
3
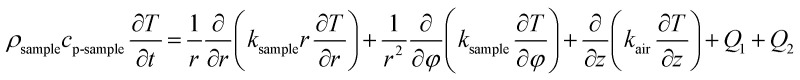


*Q*_1_ is the chemical reaction heat of the sample, W m^−3^, and *Q*_2_ is the built-in heating source of the reactor, W m^−3^. The composite boundary conditions of the whole heat transfer region are as follows:4
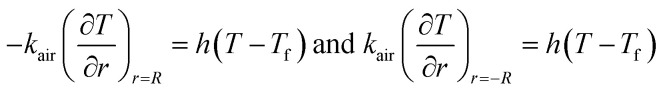
5
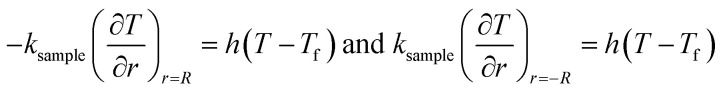
6
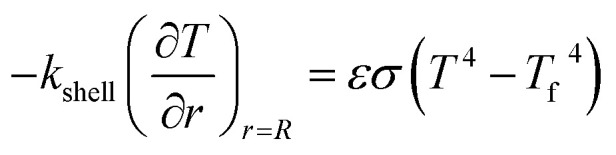


The initial conditions of the calculation are as follows:

7 *T*(*r*,*t*) = *T*_0_, at *t* = 0

In order to speed up the convergence, the 1st-order upwind differencing scheme was used to discretize the spatial-derivative term. Meanwhile, a fully implicit scheme was employed to discretize the transient term. The heat sources were customized by using the user-defined functions (UDFs) available in FLUENT. The numerical elements of the samples and air regions were hexahedral. The numerical elements have a volume of about 1.10 × 10^−8^ m^3^ to 1.16 × 10^−8^ m^3^ and the total number of numerical meshes is 39552. Grid-independence tests were conducted to guarantee that the mesh employed gave calculation results of adequate accuracy.

## 3. Results and discussion

### 3.1 Microstructural characterization of magnesium sulfate based thermochemical materials

**Fig. 2:**
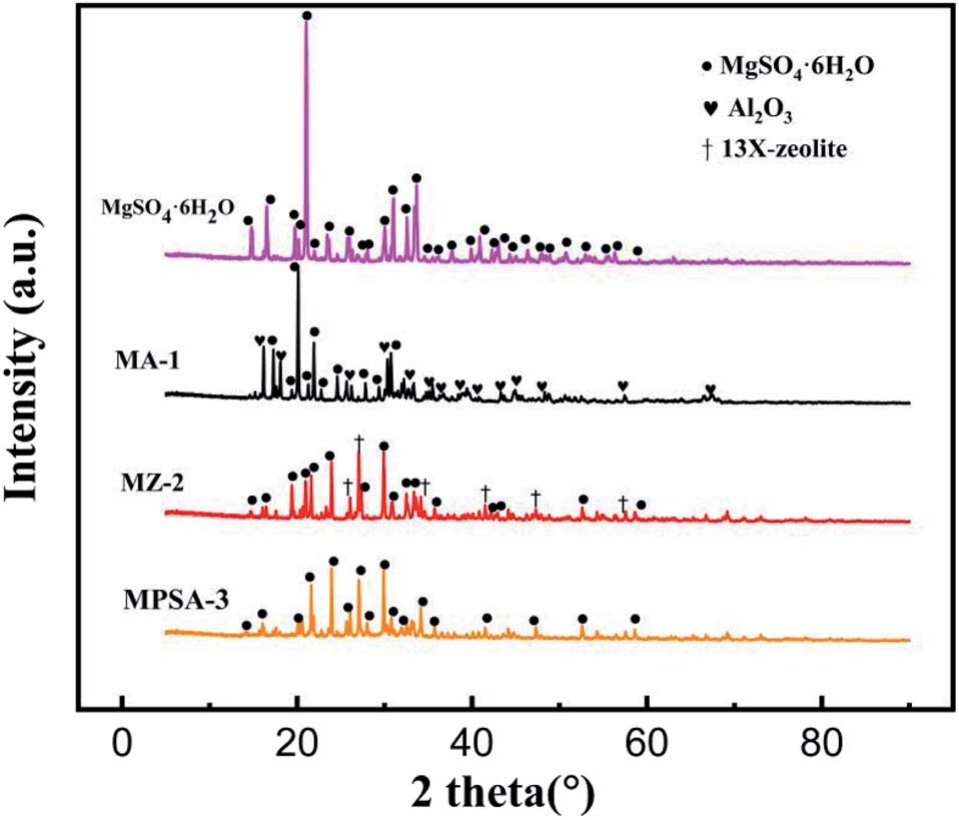
XRD patterns of thermochemical heat storage materials composed of MgSO_4_·6H_2_O, MA-1 (MgSO_4_·6H_2_O/Al_2_O_3_), MZ-2 (MgSO_4_·6H_2_O/13X-zeolite) and MPSA-3 (MgSO_4_·6H_2_O/poly(sodium acrylate)).

Fig. 2 shows the XRD spectra of the MgSO_4_·6H_2_O, MA-1, MZ-2 and MPSA-3 samples. Table 2 shows the locations of the X-ray diffraction peak of the different materials observed in Fig. 2. The diffraction peaks at around 14.7° to 58.7° (Table 2) are attributed to MgSO_4_·6H_2_O (PDF# 24-0719). And the diffraction peaks at around 16.2° to 67.5° (Table 2) are assigned to Al_2_O_3_. 13X-zeolite could be identified in MZ-2 by six diffraction peaks at around 26.0°, 26.8°, 34.5°, 41.4°, 47.3° and 57.4° (Table 2). But there is no diffraction peak for poly(sodium acrylate) in sample MPSA-3. This may be because poly(sodium acrylate)the exists in a noncrystalline state, and in XRD characterization, only crystals have a diffraction effect on X-rays. As shown in Fig. 2, the high-strength diffraction peaks of pure MgSO_4_·6H_2_O are sharp and strong. Meanwhile, comparing the intensity of diffraction peaks of MgSO_4_·6H_2_O in the composite materials (MA-1, MZ-2, MPSA-3), it was found that the diffraction intensity was similar to those of MgSO_4_·6H_2_O in MZ-2 and MPSA-3. The intensities of the MgSO_4_·6H_2_O diffraction peak of these two composite materials were slightly lower than that in MA-1. And in all these composite materials, the intensity of the MgSO_4_·6H_2_O diffraction peak was significantly weaker than that of pure MgSO_4_·6H_2_O. This indicates the successful and good dispersion of MgSO_4_·6H_2_O in the composite materials.

**Table 2:** Location of X-ray diffraction peaks of the different materials observed in Fig. 2

Materials	Diffraction peak location/2*θ*°
MgSO_4_·6H_2_O	14.7°, 16.5°, 19.7°, 20.2°, 21.0°, 21.9°, 23.4°, 25.7°, 28.0°, 30.0°, 31.0°, 32.5°, 33.6°, 34.5°, 36.1°, 37.7°, 39.8°, 40.8°, 42.3°, 43.0°, 44.2°, 46.3°, 47.8°, 48.8°, 50.6°, 52.5°, 55.5°, 56.3°, 58.7°
13X-zeolite	26.0°, 26.8°, 34.5°, 41.4°, 47.3°, 57.4°
Al_2_O_3_	16.2°, 18.1°, 25.6°, 30.3°, 32.9°, 35.4°, 36.7°, 38.9°, 40.5°, 43.4°, 44.9°, 48.5°, 57.3°, 67.5°

**Fig. 3:**
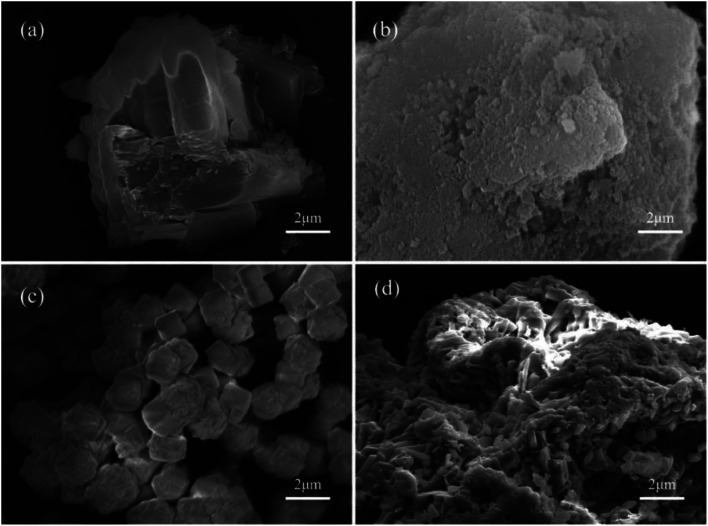
SEM images of thermochemical heat storage materials composed of (a) MgSO_4_·6H_2_O, (b) MA-1 (MgSO_4_·6H_2_O/Al_2_O_3_), (c) MZ-2 (MgSO_4_·6H_2_O/13X-zeolite) and (d) MPSA-3 (MgSO_4_·6H_2_O/poly(sodium acrylate)).

Fig. 3a–d provide the SEM images of thermochemical heat storage materials composed of MgSO_4_·6H_2_O, MA-1, MZ-2 and MPSA-3. From the SEM characterization, a big bulk MgSO_4_·6H_2_O crystal existing in the form of stacked flakes (Fig. 3a) with a diameter of around 6 μm could be clearly seen. And after composition with Al_2_O_3_, 13X-zeolite and poly(sodium acrylate) (Fig. 3b–d), the minimum diameter of MgSO_4_·6H_2_O could reach 200–500 nm. MgSO_4_·6H_2_O particles were well dispersed according to the XRD results, but some parts of the hygroscopic additives, especially the surface (Fig. 3d), were covered. This may affect the heat and mass transfer property during the heat discharge process. The MgSO_4_·6H_2_O content of MA-1, MZ-2, MPSA-3 is about 56%. During the preparation process the hygroscopic additives could retard the aggregation of MgSO_4_·6H_2_O. The surface texture of MgSO_4_·6H_2_O, MA-1, MZ-2 and MPSA-3 were tested by nitrogen adsorption–desorption. The BET specific surface area, pore volume and average pore size are provided in Table 3. These textural parameters were automatically obtained from the nitrogen adsorption–desorption measurements, which were carried out on a Quantachrome QDS-30 analyzer. The specific surface area value was obtained based on the equation: *S*_w_ = *V*_m_ × *λN*/*V*_0_ (BET method);^36^ where *S*_w_ is the specific surface area; *V*_m_ is the monolayer adsorption volume in the standard state; *λ* is the adsorbate molecular cross-sectional area, where the adsorbate molecule here is nitrogen gas; *λ* = 0.162 nm^2^; *N* is the Avogadro constant (6.02 × 10^23^); and *V*_0_ is the standard molar volume of adsorbate (22.4 cm^3^ mol^−1^). The pore volume was obtained by a single-point adsorption process. The average pore size was obtained from the BJH method.^37^ Because of the introduction of different hygroscopic additives, the specific surface areas of MA-1 (213 m^2^ g^−1^) and MZ-2 (281 m^2^ g^−1^) are higher than those of MPSA-3 (65 m^2^ g^−1^) and MgSO_4_·6H_2_O (16 m^2^ g^−1^). Combined with the SEM and XRD characterization results, it could be concluded that high specific surface area is another important factor for the nanoscale dispersion of MgSO_4_·6H_2_O.

**Table 3:** Textural parameters of hygroscopic nanoadditive modified thermochemical materials

Samples	Surface area (m^2^ g^−1^)	Pore volume (mL g^−1^)	Average pore size (nm)
MA-1	213	0.19	2.19
MZ-2	281	0.16	2.37
MPSA-3	65	0.02	1.51
MgSO_4_·6H_2_O	16	0.04	2.81

### 3.2 Heat storage performance testing of magnesium sulfate based thermochemical materials

**Fig. 4:**
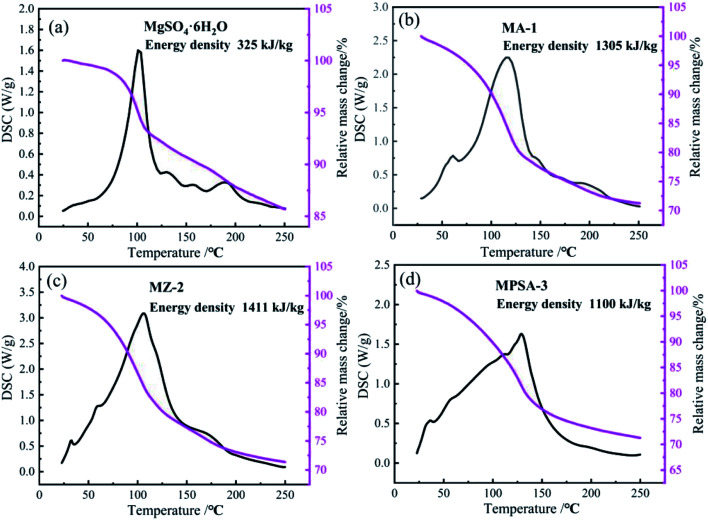
TG-DSC curves of the samples: (a) MgSO_4_·6H_2_O, (b) MA-1 (MgSO_4_·6H_2_O/Al_2_O_3_), (c) MZ-2 (MgSO_4_·6H_2_O/13X-zeolite) and (d) MPSA-3 (MgSO_4_·6H_2_O/poly(sodium acrylate)) after 30 min of hydration.

The results of the heat storage performance tests of MgSO_4_·6H_2_O, MA-1, MZ-2 and MPSA-3 are shown in Fig. 4. The related reaction is MgSO_4_·6H_2_O = MgSO_4_ + 6H_2_O. The conversion rate is lower for pure magnesium sulfate, which is only 28% after 30 min of hydration. And the reaction heat of MgSO_4_·6H_2_O is only about 325 kJ kg^−1^ (Fig. 4a). Fig. 4b shows the heat storage performance of Al_2_O_3_-promoted MgSO_4_·6H_2_O. It can be seen that MgSO_4_ has completely reacted and the energy density of MA-1 could reach 1305 kJ kg^−1^. The energy density of MZ-2 rises to a higher value (1411 kJ kg^−1^, Fig. 4c) compared to the poly(sodium acrylate) modified MgSO_4_·6H_2_O sample MPSA-3 (1100 kJ kg^−1^, Fig. 4d). The conversion reactions of MA-1, MZ-2 and MPSA-3 are fully completed. The respective conversion rates reach 100%, which are much higher than for pure magnesium sulfate. Compared with previous research,^24,26^ when 13X-zeolite is used as the additive in this work, the energy density of MZ-2 (MgSO_4_·6H_2_O/13X-zeolite; 1411 kJ kg^−1^) could be 1.3 and 2.2 times higher than in previous research (1090 kJ kg^−1^; 648 kJ kg^−1^; magnesium sulfate content: 15%; impregnation method), reported in ref. 24 and 26, respectively. These results could be attributed to the materials preparation method and higher content of MgSO_4_·6H_2_O (56%) in the composite materials. In this work, a hydrothermal method is used to prepare the composite materials and with this approach the material particles generated could be smaller and the mixed ratio could be well controlled. The heat storage performance test indicates that drawing poly(sodium acrylate), 13X-zeolite and nano-Al_2_O_3_ into magnesium sulfate hexahydrate gives a dramatically enhanced reaction rate for MgSO_4_ and water vapor under the same hydration reaction conditions. The reason lies in the hygroscopic properties of the additives that make H_2_O adsorption easier so more water molecules come into contact with the reaction interface on MgSO_4_. The reason for the higher energy density of the modified materials is the higher specific surface area (Table 3), which improves the particle dispersion of MgSO_4_·6H_2_O and enlarges the contact area for water molecules. When the particle size is reduced to the nanoscale, the surface atoms would notably increase and a large number of dangling bonds would be formed, which could lead to improved thermodynamic properties.^38,39^ It could be concluded that small nanoparticles could provide a greater contribution to energy density enhancement.

**Fig. 5:**
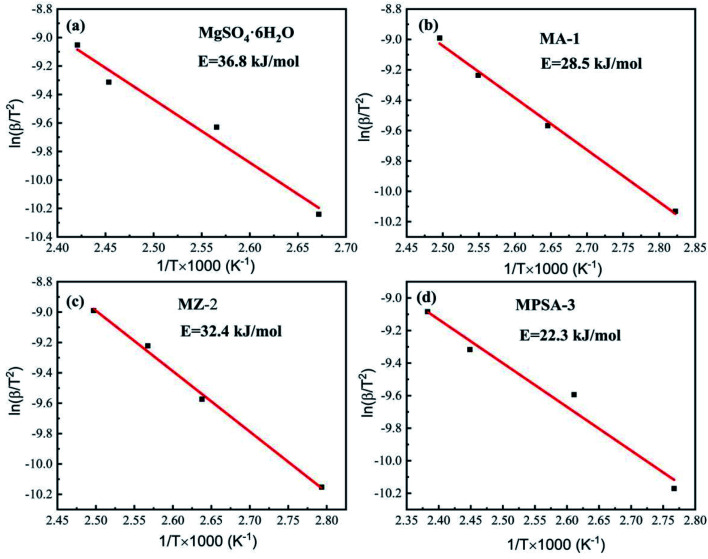
Activation energy of the dehydration reaction of (a) MgSO_4_·6H_2_O, (b) MA-1 (MgSO_4_·6H_2_O/Al_2_O_3_), (c) MZ-2 (MgSO_4_·6H_2_O/13X-zeolite) and (d) MPSA-3 (MgSO_4_·6H_2_O/poly(sodium acrylate)).

Tables 4–7 show the DSC analysis and results of the kinetic parameter calculations for MgSO_4_·6H_2_O, MA-1, MZ-2 and MPSA-3 that include the heating rate, peak temperature and related functions. Fig. 5a–d show the linear fitting curve of the modified Arrhenius's equation and the reaction activation energy, which is calculated by the Kissinger method.^35^ For MgSO_4_·6H_2_O the decomposition activation energy is the highest among the synthesized thermochemical materials and reaches 36.8 kJ mol^−1^ (Fig. 5a). However, hygroscopic additives make the endothermic reaction easier and decrease the activation energy (Fig. 5b–d). After adding Al_2_O_3_, 13X-zeolite and poly(sodium acrylate), the activation energies decrease to 28.5 kJ mol^−1^, 32.4 kJ mol^−1^ and 22.3 kJ mol^−1^, respectively. This proves that the addition of hygroscopic additives could remarkably decrease the difficulty of the heat storage reaction. According to the SEM results in Fig. 3a–d, this reduction in activation energy may relate to the change in particle size of MgSO_4_·6H_2_O. As the MgSO_4_·6H_2_O particle size becomes smaller from big bulk (Fig. 3a) to nanoparticles (Fig. 3b–d) the average value of the activation energy becomes about 0.75 times lower than that of MgSO_4_·6H_2_O. It can be inferred that the microstructure and kinetic behavior have some relationship and the reaction activation energy is an important parameter for the heat storage reaction and could change perceptibly with different materials.

**Table 4:** DSC analysis and the results of the kinetic parameter calculations of MgSO_4_·6H_2_O

Heating rate *β* (K min^−1^)	Peak temperature *T* (K)	1/*T* × 1000 (K^−1^)	ln *β*/*T*^2^
5	374.3	2.67165	−10.24068
10	389.8	2.56542	−9.62868
15	407.6	2.45339	−9.31252
20	413.1	2.42072	−9.05165

**Table 5:** DSC analysis and results of the kinetic parameter calculations of MA-1 (MgSO_4_·6H_2_O/Al_2_O_3_)

Heating rate *β* (K min^−1^)	Peak temperature *T* (K)	1/*T* × 1000 (K^−1^)	ln *β*/*T*^2^
5	354.3	2.82247	−10.13085
10	378.0	2.64550	−9.56720
15	392.3	2.54907	−9.23600
20	400.7	2.49563	−8.99069

**Table 6:** DSC analysis and results of the kinetic parameter calculations of MZ-2 (MgSO_4_·6H_2_O/13X-zeolite)

Heating rate *β* (K min^−1^)	Peak temperature *T* (K)	1/*T* × 1000 (K^−1^)	ln *β*/*T*^2^
5	358.0	2.79330	−10.15163
10	379.1	2.63783	−9.57301
15	389.5	2.56739	−9.22168
20	400.5	2.49688	−8.98970

**Table 7:** DSC analysis and results of the kinetic parameter calculation for MPSA-3 (MgSO_4_·6H_2_O/poly(sodium acrylate))

Heating rate *β* (K min^−1^)	Peak temperature *T* (K)	1/*T* × 1000 (K^−1^)	ln *β*/*T*^2^
5	361.4	2.76702	−10.17053
10	383.0	2.61097	−9.59348
15	408.4	2.44858	−9.31644
20	419.7	2.38265	−9.08335

**Fig. 6:**
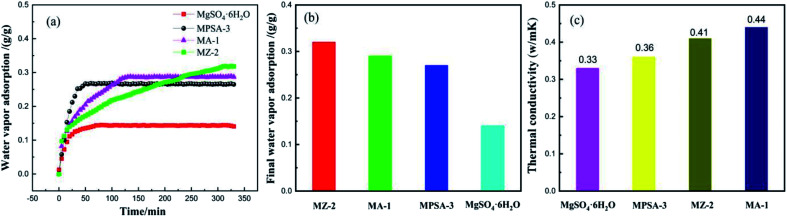
(a) Water vapor adsorption; (b) the saturated adsorption amount; (c) thermal conductivity of MgSO_4_·6H_2_O, MA-1 (MgSO_4_·6H_2_O/Al_2_O_3_), MZ-2 (MgSO_4_·6H_2_O/13X-zeolite) and MPSA-3 (MgSO_4_·6H_2_O/poly(sodium acrylate)).

Fig. 6a and b show the water vapor adsorption testing of the mass transfer properties of MgSO_4_·6H_2_O, MA-1, MZ-2 and MPSA-3, respectively. The adsorption curves of all of the samples show rapid rises for the stages of water adsorption and saturated water adsorption. These two stages exactly correspond to the water vapor mass transfer during the hydration reaction, which is attributed to physical/chemical adsorption accompanied by an exothermic and rapid increase in water adsorption and the adsorption balance. The water vapor saturated adsorption amount of MZ-2 is the highest among the samples (Fig. 6b), and the adsorption rate is also higher (Fig. 6a). This may be due to two factors: the hygroscopic porous structure of the 13X-zeolite and the nano-dispersion of MgSO_4_·6H_2_O. All these factors are advantageous to water adsorption. When hygroscopic additives are composited with MgSO_4_·6H_2_O, the adsorption rate is obviously affected. The adsorption rate of the poly(sodium acrylate) modified composite material MPSA-3 is the highest. Furthermore, highly dispersed MgSO_4_·6H_2_O provides enhanced water vapor adsorption. The composited material MZ-2 shows high water vapor adsorption (almost twice the adsorption amount of pure MgSO_4_·6H_2_O, Fig. 6b) even though the content is only about half (56%). Enhanced water adsorption exhibits efficient mass transfer, which will improve the hydration reaction. For the thermal conductivity (Fig. 6c), adding Al_2_O_3_, 13X-zeolite and poly(sodium acrylate) could also change the heat transfer property of MgSO_4_·6H_2_O, and meet the requirement for efficient utilization of thermal energy. Now, in order to promote the matching degree and future application of these materials, the heat transfer behavior of the MgSO_4_-based composite heat storage materials in stainless steel reactor was simulated.

### 3.3 Numerical simulations for heat transfer behavior of magnesium sulfate based composite heat storage material in the reactor

**Fig. 7:**
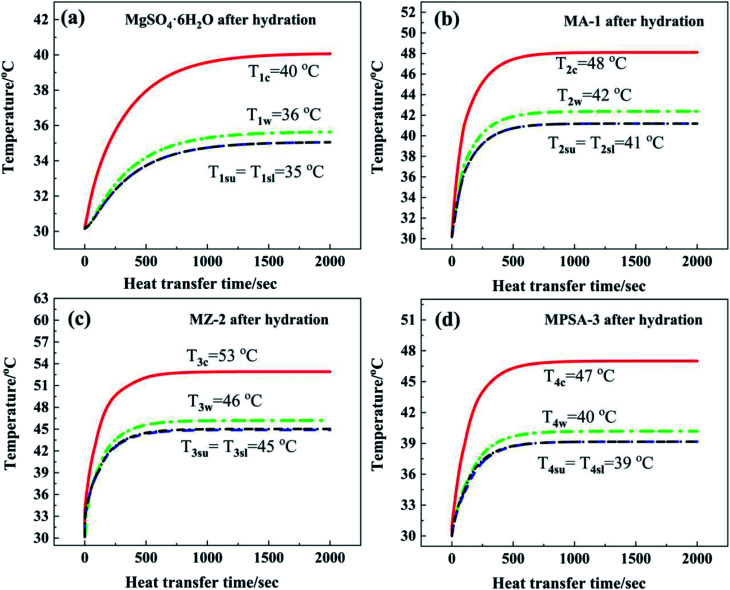
Numerical simulations of temperature–time curves in the reactor: (a) MgSO_4_·6H_2_O, (b) MA-1 (MgSO_4_·6H_2_O/Al_2_O_3_), (c) MZ-2 (MgSO_4_·6H_2_O/13X-zeolite) and (d) MPSA-3 (MgSO_4_·6H_2_O/poly(sodium acrylate)) after the hydration reaction.

Fig. 7a–d show the numerical simulation temperature curves of the contact face in different areas of the reactor when the heat storage materials MgSO_4_·6H_2_O, MA-1, MZ-2 and MPSA-3 are respectively packed in the reactor. All the temperature–time curves show a rapid upward trend in the initial temperature and a stable final temperature. This reflects the heat transfer process in the reactor, which consists of two stages. In the first stage the exothermic heat of reaction is higher than the heat elimination by the environment, leading to an endothermic and quick temperature rise of the reactor. The second is the heat balance stage: the exothermic heat of reaction is equal to the heat dissipation by the environment, meaning there is no temperature change.

Fig. 7a shows the core temperature *T*_1c_ of MgSO_4_·6H_2_O (40 °C) in the center of the reactor. The core temperature is higher than the temperature in other positions of the reactor, which is due to the lower thermal conductivity of the materials. Under the same heat dissipation conditions, this causes higher thermal resistance in the direction of reaction heat transferred along the perpendicular from the reactor center to the inner wall. The inner wall temperature *T*_1w_ in the reactor was 36 °C. The difference between temperatures *T*_1c_ and *T*_1w_ was 4 °C. By comparison, the temperature gradients from the reactor center to the lower surface and upper surface of the heat storage material were a little higher than that from the reactor center to the inner wall (difference in temperature between *T*_1c_ and *T*_1su_ and *T*_1sl_: 5 °C). The characteristic temperature distribution in the reactor was a high-temperature area in the center of the reactor and a low-temperature area near the reactor wall. The average temperature *T*_1sl_ of the interface between N_2_ gas with water vapor and MgSO_4_·6H_2_O at the lower surface (35 °C) was equal to the average temperature *T*_1su_ of the upper surface (35 °C). After 1500 s the reactor could reach heat balance and the temperature stayed the same. In summary, the heat dissipation capacity of the bottom layer material was as good as that of the upper layer, and the released thermal energy was mainly transferred through the side wall of the reactor.

Fig. 7b–d show the temperature–time curves of the interface between the hygroscopic additive modified heat storage materials MA-1, MZ-2 and MPSA-3 and the reactor, respectively. It can be observed that the temperature of the material center and the contact surface between the heat storage material and the reactor could reach a heat balance when the reactions were sustained for about 850 s, 880 s, and 1000 s, respectively (Fig. 7b, c and d). Compared with the pure material MgSO_4_·6H_2_O (where the time required for heat balance is 1500 s, Fig. 7a), the composite material takes less time to reach heat balance, the heat transfer rate is faster and the exothermic temperature is higher than those of MgSO_4_·6H_2_O. Among them, the core temperature *T*_3c_ of MZ-2 (Fig. 7c) is the highest, which can reach 53 °C, 13 °C higher than that of MgSO_4_·6H_2_O. The reactor inner wall temperature *T*_3w_ is 10 °C higher than that of MgSO_4_·6H_2_O (Fig. 7a). The temperature at the lower surface (*T*_3sl_) and upper surface (*T*_3su_) of MZ-2 is also 10 °C higher than that of MgSO_4_·6H_2_O. The exothermic temperatures at the heat balance stage in different interfaces of the other two materials are MA-1: *T*_2c_ = 48 °C, *T*_2w_ = 42 °C, *T*_2sl_ = *T*_2su_ = 41 °C (Fig. 7b) and MPSA-3: *T*_4c_ = 47 °C, *T*_4w_ = 40 °C, *T*_4sl_ = *T*_4su_ = 39 °C (Fig. 7d).

**Fig. 8:**
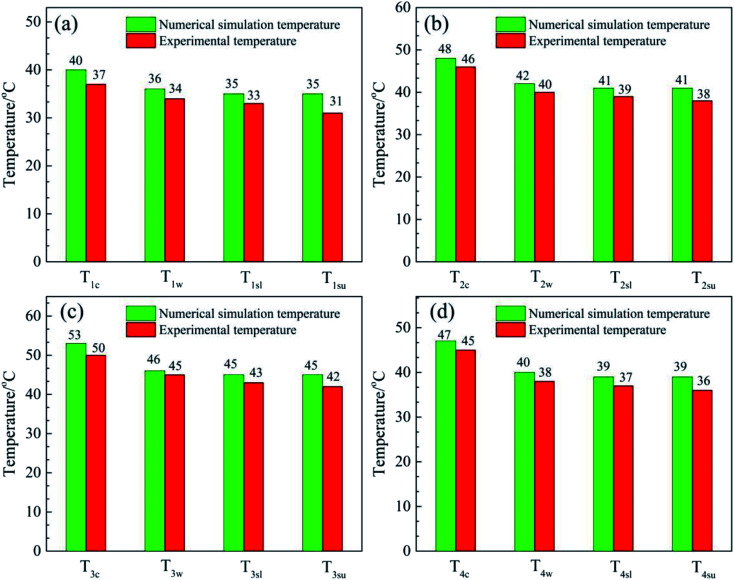
Comparison of the numerical simulation and experimental temperature data for heat storage materials in the reactor: (a) MgSO_4_·6H_2_O, (b) MA-1 (MgSO_4_·6H_2_O/Al_2_O_3_), (c) MZ-2 (MgSO_4_·6H_2_O/13X-zeolite) and (d) MPSA-3 (MgSO_4_·6H_2_O/poly(sodium acrylate)) after the hydration reaction.

Fig. 8 shows a comparison of the numerical simulation and experimental temperature data for heat storage materials in the reactor. It can be seen that the simulated and experimental temperatures (*T*_c_, *T*_w_, *T*_sl_ and *T*_su_) of the materials are almost equivalent. This indicates that the numerical simulation of the heat transfer process was accurate and extremely valuable for future material and reactor design. The results of the numerical simulation of the heat transfer process prove that the heat release and heat transfer properties of the hygroscopic additive modified composites (MA-1, MZ-2 and MPSA-3) are better than those of the pure material (MgSO_4_·6H_2_O) in a stainless steel reactor.

**Fig. 9:**
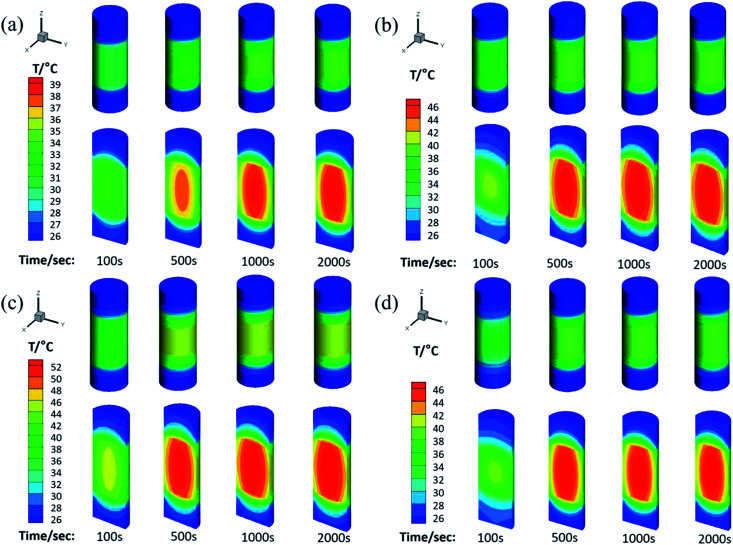
The temperature distribution for heat storage materials in the reactor: (a) MgSO_4_·6H_2_O, (b) MA-1 (MgSO_4_·6H_2_O/Al_2_O_3_), (c) MZ-2 (MgSO_4_·6H_2_O/13X-zeolite) and (d) MPSA-3 (MgSO_4_·6H_2_O/poly(sodium acrylate)) after the hydration reaction.

The physical model used for the chemical heat storage material is a cylindrical stainless-steel reactor (Fig. 1a). On the basis of which, the heat transfer process is investigated and a numerical calculation mesh 3D model is established (Fig. 1b). And then the energy conservation equation is solved based on the finite volume method to simulate the heat transfer process in the reactor. Fig. 9 shows the temperature distribution for heat storage materials in the reactor for (a) MgSO_4_·6H_2_O, (b) MA-1, (c) MZ-2 and (d) MPSA-3 after the hydration reaction. It can be seen from Fig. 9a–d that as the reaction progressed for 100 seconds, the temperature in the center of the reactor where the sample was located gradually rose and could reach above 40 °C except for MgSO_4_·6H_2_O. Because of the low thermal conductivity and heat concentration of the materials, the central heating rate of the samples was faster and the core temperature was higher than those of other interfaces in the reactor. As the reaction proceeded and after 500 s the heat was gradually released in the reactor and started to transfer from the center of the heat storage material to the surroundings, which caused a rearrangement of the temperature and finally reached heat balance. And the temperature of the hygroscopic additive modified composited materials rose faster than that of the pure material. The whole temperature evolution showed the dynamic change in temperature in the heat release process of the reactor, which provided an advanced strategy for thermal energy utilization and the subsequent synthesis of heat storage materials and the optimal design of the reactor.

## 4. Conclusions

In this paper, a hydrothermal method was used to synthesize a hygroscopic additive: super-absorbent polymer material (poly(sodium acrylate)), 13X-zeolite and nano-aluminum oxide (nano-Al_2_O_3_) modified magnesium sulfate hexahydrate (MgSO_4_·6H_2_O) composite thermochemical materials for low-temperature heat storage. After being composed with 13X-zeolite, nano-Al_2_O_3_ and poly(sodium acrylate), MgSO_4_·6H_2_O crystals are dispersed into nanoparticles (200–500 nm). The introduction of hygroscopic materials leads to a distinct decrease in activation energy for the heat storage reaction and an obvious increase in heat storage performance because of the excellent water adsorption properties and dispersal effect of the hygroscopic additives. The initial activation energy value and energy density of MgSO_4_·6H_2_O are 36.8 kJ mol^−1^ and 325 kJ kg^−1^, respectively. For nano-Al_2_O_3_ modified composite material MA-1, the activation energy reaches 28.5 kJ mol^−1^ and the heat storage energy density is 1305 kJ kg^−1^. But after modification by poly(sodium acrylate), the activation energy could decrease to 22.3 kJ mol^−1^ (MPSA-3). This indicates that the activation energy can be reduced by expanding the specific surface area or improving the hydrophilicity of materials. Among the prepared materials, super-absorbent polymer material (poly(sodium acrylate)) modified composite material MPSA-3 shows a good heat storage energy density (1100 kJ kg^−1^) and the lowest activation energy. 13X-zeolite modified composite material MZ-2 shows a lower activation energy (32.4 kJ mol^−1^) and the highest heat storage energy density (1411 kJ kg^−1^), which is 4.3 times higher than that of pure magnesium sulfate hexahydrate. According to a numerical simulation of the heat transfer, the involvement of hygroscopic additives could greatly change the temperature distribution in the reactor and efficiently export thermal energy to the outside thermal load side. The temperature values of experimental and numerical simulation are similar. This proves that the result of the numerical simulation is very close to the actual heat transfer behavior. This energy storage system could output thermal energy at around 50 °C and absorb heat in the range of 100–200 °C. This research proposes an advanced strategy combining thermochemical nanomaterials preparation followed by a material-reactor heat transfer numerical simulation, which will provide strong support for future materials and reactor design in the field of low-temperature thermal energy storage. Based on this research, the focus of the next step should be the size-controlled preparation of thermochemical materials and an investigation of the heat transfer behavior at the micro- and nanoscale and reactor design.

## Author contributions

This research was conceived and designed with the participation of all the authors. Shijie Li and Xiangyu Yang contributed equally to this work. Shijie Li: conceptualization, writing – original draft, funding acquisition; Xiangyu Yang: resources; data curation; writing – review & editing; Lisheng Deng and Yongchun Fu: visualization, formal analysis; Mingjun Pang and Ti Dong: validation; Lingna Sua: investigation, Shang Jiang and Yisong Yu: supervision and funding acquisition. All authors have read and agreed to the published version of the manuscript.

## Nomenclature

*C*_p_
: Specific heat capacity (J kg^−1^ K^−1^)
ρ
: Gas density (kg m^−3^)
T
: Temperature (K)
t
: Time (s)
k
: Thermal conductivity coefficients (W m^−1^ K^−1^)
r
: Cylinder material model radius (m)
φ
: Central angle
z
: Cylinder material model height (m)
R
: Reactor radius (m)
ε
: Emissivity
σ
: Stefan–Boltzmann's constant (W m^−2^ K^−4^)
f
: Outside low temperature object
E
: Activation energy (kJ mol^−1^)
*T*_1c_
: Core temperature of MgSO_4_·6H_2_O in reactor (°C)
*T*_1w_
: Inner wall temperature of reactor with MgSO_4_·6H_2_O (°C)
*T*_1sl_
: Lower surface temperature of MgSO_4_·6H_2_O in reactor (°C)
*T*_1su_
: Upper surface temperature of MgSO_4_·6H_2_O in reactor (°C)
*T*_2c_
: Core temperature of MA-1 in reactor MA-1 (°C)
*T*_2w_
: Inner wall temperature of reactor with MA-1 (°C)
*T*_2sl_
: Lower surface temperature of MA-1 in reactor (°C)
*T*_2su_
: Upper surface temperature of MA-1 in reactor (°C)
ϕ
: Reactor diameter (mm)
h
: Reactor height (mm)
β
: Heating rate (K min^−1^)
R
: Molar gas constant (J mol^−1^ K^−1^) in Arrhenius's equation
A
: Pre-exponential factor
α
: Dehydration conversion rate
*T*_c_
: Core temperature of material in reactor (°C)
*T*_w_
: Inner wall temperature of reactor (°C)
*T*_sl_
: Lower surface temperature of material in reactor (°C)
*T*_su_
: Upper surface temperature of material in reactor (°C)
*S*_w_
: Specific surface area (m^2^ g^−1^)
*V*_m_
: Monolayer adsorption volume at standard state (cm^3^)
λ
: Adsorbate molecular cross-sectional area (nm^2^)
N
: Avogadro constant (6.02 × 10^23^)
*V*_0_
: Standard molar volume of adsorbate (22.4 cm^3^ mol^−1^)
*T*_3c_
: Core temperature of MZ-2 in reactor (°C)
*T*_3w_
: Inner wall temperature of reactor with MZ-2 (°C)
*T*_3sl_
: Lower surface temperature of MZ-2 in reactor (°C)
*T*_3su_
: Upper surface temperature of MZ-2 in reactor (°C)
*T*_4c_
: Core temperature of MPSA-3 in reactor (°C)
*T*_4w_
: Inner wall temperature of reactor with MPSA-3 (°C)
*T*_4sl_
: Lower surface temperature of MPSA-3 in reactor (°C)
*T*_4su_
: Upper surface temperature of MPSA-3 in reactor (°C)
w
: Reactor wall thickness (mm)

## Conflicts of interest

There are no conflicts to declare.

## Supplementary Material
